# Environmental DNA from peck marks shows potential for non‑invasive monitoring of woodpeckers

**DOI:** 10.1371/journal.pone.0328831

**Published:** 2025-08-20

**Authors:** Muhammad Bilal Sharif, Björn Ferry, Jérôme Fuchs, Bodil Cronholm, Peter D. Heintzman, Love Dalén

**Affiliations:** 1 Centre for Palaeogenetics, Stockholm, Sweden; 2 Department of Zoology, Stockholm University, Stockholm, Sweden; 3 Independent Researcher, Storuman, Sweden; 4 Institut de Systématique Evolution Biodiversité, Muséum national d’Histoire naturelle CNRS SU EPHE UA, Paris, France; 5 Department of Bioinformatics and Genetics, Swedish Museum of Natural History, Stockholm, Sweden; 6 Department of Geological Sciences, Stockholm University, Stockholm, Sweden; Institute of Systematics and Evolution of Animals Polish Academy of Sciences, POLAND

## Abstract

Monitoring species’ occurrences is essential for understanding ecosystem dynamics, tracking biodiversity changes, and guiding conservation efforts. Traditional monitoring methods, such as visual surveys, are challenging, particularly for elusive and endangered species. This proof-of-concept study explores the potential of environmental DNA (eDNA) collected from peck marks as a non-invasive tool for detecting and identifying woodpecker species. We collected nine samples from fresh peck marks on birch and spruce trees in the forests of Swedish Lapland. In two samples, we successfully amplified an 81 base-pair fragment of the woodpecker mitochondrial 16S rRNA gene. Taxonomic assignment identified the Eurasian three-toed woodpecker (*Picoides tridactylus*), a species classified as “Near Threatened” in Sweden. We collected an additional 15 samples from 4-19 years old peck marks preserved inside the trunks of birch and pine trees in the same area. No woodpecker DNA was detected in these samples, likely due to DNA degradation. Our findings demonstrate the potential of using eDNA from peck marks as a non-invasive approach for monitoring elusive woodpecker species.

## Introduction

Monitoring the occurrences and abundance of species is essential for making informed decisions for habitat protection and biodiversity conservation. The tracking of species distributions through time improves understanding of ecosystem dynamics, biodiversity changes, and the effects of external environmental changes. These monitoring efforts help conservation managers to identify critical habitats necessary for ensuring population viability [1], thereby enabling authorities to prioritize areas for intervention, whether through policy changes or direct conservation actions, while providing a framework to evaluate the effectiveness of existing conservation strategies.

Species monitoring is crucial for endangered taxa. However, by definition, endangered species are rare and often elusive, making direct visual observations especially challenging. Consequently, conservation managers often rely on alternative methods, such as analyzing tracks or faeces, employing camera traps or acoustic sensors, or detecting other indirect traces left by species during foraging and other activities. One example of such use of indirect traces in species monitoring comes from the woodpecker family Picidae, where characteristic peck marks and holes made in trees have been used to detect the presence of various species [2–4]. For example, the Eurasian three-toed woodpecker (*Picoides tridactylus*) makes a series of holes (sometimes referred to as “sap rings” or “sap rows”) around tree trunks to access nutrient-rich sap during the spring. In Sweden, where eight extant species of woodpecker exist (Table 1), the presence of these sap rows, particularly on spruce and pine trees, has been suggested as an indicator of the Eurasian three-toed woodpecker [2,3]. Similarly, the critically endangered White-backed woodpecker (*Dendrocopos leucotos*) in Sweden has also been suggested to leave distinctive foraging holes, which can serve as useful indicators for its presence during surveys [4]. However, these sap rows and foraging holes are not necessarily species-specific. Similar sap rows have also been reported from other woodpecker species in both Eurasia and North America [5,6]. Among these, the Great spotted woodpecker (*Dendrocopos major*), Black woodpecker (*Dryocopus martius*), and European green woodpecker (*Picus viridis*) are sympatric with the Eurasian three-toed woodpecker in Sweden [3]. This is important because the presence of an endangered woodpecker species has a direct impact on local forestry practices. For example, when the Eurasian three-toed or White-backed woodpecker is detected in an area, the Swedish Forest Agency issues specific guidelines on forestry operations, including recommendations on the timing and methods of logging. These measures aim to minimize disruption to critical habitats, highlighting the importance of species monitoring in balancing conservation and sustainable land use. Given these challenges, additional methods are needed to accurately detect and identify specific woodpecker species in an area.

**Table 1:** Swedish woodpecker species.

Common Name	Scientific Name	Swedish Red List Status
Eurasian Wryneck	*Jynx torquilla*	Least Concern
European Green Woodpecker	*Picus viridis*	Least Concern
Great Spotted Woodpecker	*Dendrocopos major*	Least Concern
Grey-headed Woodpecker	*Picus canus*	Least Concern
Black Woodpecker	*Dryocopus martius*	Near Threatened
Eurasian Three-toed Woodpecker	*Picoides tridactylus*	Near Threatened
Lesser Spotted Woodpecker	*Dryobates minor*	Near Threatened
White-backed Woodpecker	*Dendrocopos leucotos*	Critically Endangered
Middle Spotted Woodpecker	*Dendrocoptes medius*	Extirpated in Sweden

Nine woodpecker species currently present or historically recorded in Sweden and their conservation status [7]

One such method is the use of environmental DNA (eDNA), which uses trace amounts of DNA shed or left behind by the organism. eDNA provides a powerful and non-invasive method for monitoring the presence of species within ecosystems [8]. Recent studies have demonstrated the utility of eDNA in detecting species presence from a variety of sources, such as scent marks [9], footprints [10], air [11], urine [12], soil [13], trees [13], deadwood [14,15], and saliva [16,17]. In this proof-of-concept study, we tested the potential of using eDNA collected from nine fresh woodpecker foraging holes for species identification. The rationale for this approach is that the act of pecking, as well as the subsequent licking of sap, should leave trace amounts of DNA, most likely from the bird’s saliva. In addition, as keratinized tissues like the beak also contain DNA [18,19], they may also contribute to the eDNA recovered from the pecking holes. Additionally, we aimed to investigate the possibility of recovering woodpecker DNA from marks inside tree trunks that represent foraging holes created several years ago, which have since been incorporated into the trunk itself. Finally, we examined a potential misannotation in the NCBI RefSeq database for the mitogenome sequence of the Eurasian Three-toed woodpecker (*Picoides tridactylus*) by incorporating seven mitogenome sequences from different woodpecker species, obtained from an ongoing phylogenetic study (Fuchs *et al.*, in prep).

## Methods

### Sample collection

**Fig 1:**
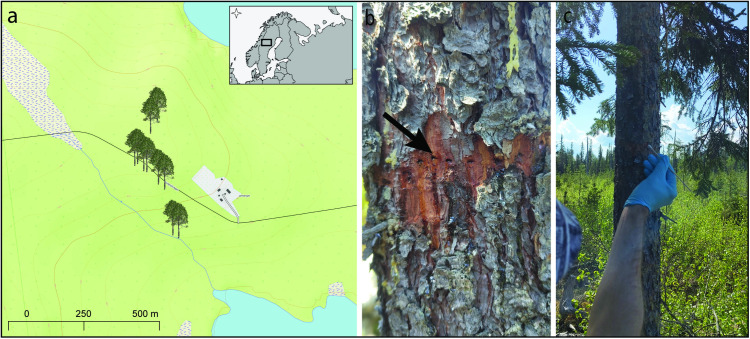
Sampling locations and eDNA collection from woodpecker peck marks. (a) Locations of the 10 trees with peck marks sampled in this study. (b) An example fresh sap row that was sampled for eDNA (photo credit: Heidi Andersson). (c) eDNA sample collection procedure using a cotton swab on a fresh sap row (photo credit: Heidi Andersson).

We searched for woodpecker peck marks on trees within a ~ 10 ha varied forest area in Swedish Lapland (latitude: 65.152877, longitude: 16.952557). We found two birch (*Betula* sp.) and two spruce (*Picea* sp.) trees with visible sap rows, indicative of recent pecking activity (at most a couple of months old). We categorized these sap rows as fresh peck marks. A sample was collected from these trees by rubbing a clean cotton swab within the sap row and then placing it into sterile biopack bags (Fig 1c). In total, nine cotton swab samples were collected from these four trees on May 29, 2024 (S1 Table). We also found additional peck marks, aged between 4 and 19 years based on growth ring counts (preserved inside the tree trunks), on four birch and two pine (*Pinus* sp.) trees in the same area. In these cases, we extracted a core sample from the tree using an increment borer (5 mm diameter, 200 mm length) and immediately stored the core in a tube prefilled with 95% ethanol. We collected 15 core samples from these 6 trees on May 2, 2024, and the equipment was disinfected in a bleach solution for five minutes between extracting each core sample (S1 Table). In total, 24 samples were taken from 10 trees located along a 500 m transect (Fig 1).

### Environmental DNA data generation

All laboratory work, up to the first amplification, was conducted in a UV cabinet within a facility designed for processing historical samples at the Swedish Museum of Natural History, Stockholm, Sweden. DNA was extracted from the fresh sap row samples using the QIAamp DNA Micro kit (QIAGEN), following the manufacturer’s protocol. Each swab was placed in a 2 mL tube, and the lower part was separated from the shaft by either breaking it against the tube wall or cutting it with a sterile scalpel. Lysis buffer and Proteinase K were then added to each tube, followed by incubation with shaking at 56 °C. For the core samples, the wood was either drilled to produce a fine powder, cut into small pieces with a scalpel, or both (S1 Table). The ethanol from the sample tube was left to evaporate for a subset of core samples and DNA extraction was performed on the remains. We used the Qiagen DNeasy Plant Mini kit (Qiagen), with 1% polyvinylpyrrolidone and 0.6 mg/mL Proteinase K added to the lysis buffer to avoid inhibitory effects on DNA extraction caused by phenolic compounds and other substances in the wood. Negative controls were included in each extraction batch for both sample types.

Amplicon libraries for Illumina sequencing were produced using a two-step PCR approach. In the first step of metabarcoding, we used two primer pairs (Aves-16S-1AF/R, Aves-16S-2AF/R) to respectively amplify ~81 bp and ~75 bp inserts of the mtDNA 16S rRNA gene (Table 2). For the first primer pair, we also included a blocking primer, Aves-16S-1A-Block, to reduce co-amplification of human contamination [20]. Both primer pairs included flanking Illumina TruSeq adapter sequences to enable the second step of indexing PCR. Metabarcoding was conducted in 25 µl reactions using Illustra™ Hot Start Mix Ready-To-Go (Cytiva), with 3 µl of DNA extract, 0.2 µM of each amplifying primer, and 2 µM of blocking primer. Cycling conditions for the PCR were 95°C for 5 min, followed by 55 cycles of 94°C for 30s, 60°C for 30s, and 72°C for 30s, and a final extension of 8 min at 72°C. We used a higher annealing temperature (60°C) compared to the original protocol [20] to increase the specificity of our PCR, as our samples likely contained DNA from multiple organisms and only very small traces of avian DNA. PCR products were visualized on an agarose gel and purified using Exonuclease I and FastAP Thermosensitive Alkaline Phosphatase (Thermo Scientific).

**Table 2:** Metabarcoding primers and Illumina adapter sequences used for woodpecker DNA amplification.

Primer Name	Sequence	Product Size
Aves-16S-1AF Aves-16S-1AR	ACACTCTTTCCCTACACGACGCTCTTCCGATCT**CATAAGACGAGAAGACCCTGTGGA** GTGACTGGAGTTCAGACGTGTGCTCTTCCGATCT**TCCAAGGTCGCCCCAACCGAA**	~193 bp
Aves-16S-2AF Aves-16S-2AR	ACACTCTTTCCCTACACGACGCTCTTCCGATCT**CCTTGGAGAAAAACAAANCCTCCAAA** GTGACTGGAGTTCAGACGTGTGCTCTTCCGATCT**TCCCTGGGGTAGCTTGGTCCAT**	~190 bp

Primers in bold, adapters in regular text.

In the second step, indexing PCR, adding unique dual indexes and the Illumina flowcell binding adapters (P5 and P7) was carried out for all products that showed a band of approximately the expected size (Table 2) and for a subset of the samples that only showed bands of unexpected sizes (S1 Table). We performed indexing PCRs in 25 µl reactions, with 0.625 U of Accuprime Pfx DNA Polymerase, 1 × AccuPrime Reaction Mix (Life Technologies, Carlsbad, CA, USA), 0.2 µM of each indexing primer and 2 µL of purified PCR product from the first PCR step. Cycling conditions for this PCR were 95°C for 2 min, followed by 7 cycles of 95°C for 15s, 55°C for 30s, and 72°C for 1 min. The amplified libraries were visualized on an agarose gel and pooled based on concentration inferred from gel band intensity. The library pool was cleaned using AMPure XP beads (Beckman Coulter) and sequenced at the National Genomics Infrastructure (Science for Life Laboratory, Stockholm, Sweden) on the NextSeq2000 platform using a P1 flowcell with 2 × 150 cycles.

The raw sequencing reads were first processed using fastp v0.24.0 [21] to merge the paired-end reads and remove the adapter and primer sequences. The merged reads were then processed and taxonomically identified using the programs implemented in OBITools package v3.0.1b26 [22]. First, identical sequencing reads were clustered together and sequences shorter than 50 bp or appearing fewer than 3 times were removed. To exclude low-frequency sequence variants, which are likely the result of PCR or sequencing errors, the reads were filtered using obiclean with a threshold of *r* (the ratio of a sequence variant’s abundance compared to the most abundant variant within a sample) set at 0.05. A custom taxonomic reference database containing all of the potentially amplifiable sequences by the primer pairs used here, was constructed using ecoPCR [23] applied to the latest snapshot of the EMBL standard sequence database (September 2024), which includes sequences from human (HUM), prokaryotic (PRO), and vertebrate (VRT) categories. The filtered reads were taxonomically assigned using the ecotag tool and matched against the custom reference database. Sequences that had an identity hit of ≥85% and were represented at least 3 times within each sample were retained for downstream analysis.

### Generating mitogenomes for Swedish woodpecker species

The amplified 16S rRNA gene sequences in our eDNA samples did not match any reference mitogenomes for Swedish woodpeckers. To investigate potential misannotations in the reference database, we complemented our eDNA dataset with seven newly generated complete mitogenomes from Fuchs et al. (in prep) (S2 Table). Here in brief, these mitogenomes were generated by extracting DNA from tissue (muscle, liver) using the Qiagen extraction kit (Qiagen, Valencia, CA) following the manufacturer’s protocol, or from a toe pad sample using a Phenol-Chloroform protocol (S2 Table). For the tissue samples, we used amplicon sequencing, where overlapping 0.8-1.5 kb fragments were amplified and Sanger-sequenced using a combination of universal and specific primers. This approach was complemented by performing overlapping long-range PCRs (4−8 kb) before being fragmented and sequenced either on an IonTorrent or Illumina Miseq platform using a mixed species pooling strategy [24]. For the toe pad sample, we used a mitochondrial bait-capture approach with a custom bait-set for Piciformes mitochondrial DNA (Fuchs *et al.* in prep). Assemblies were made using a mixed strategy of *de novo* assemblies of the reads from both platforms in Mitobim-1.9.1 (https://github.com/chrishah/MITObim [25]), NOVOPlasty-2.7.2 [26], and, if required, read mapping in *Ugene* [27]. This newly generated dataset was supplemented with a recently published mitogenome from the European green woodpecker [28], and nine other previously available mitogenomes from seven Swedish woodpecker species (accession numbers are provided in S3 Table).

To evaluate whether differences between the newly generated sequence for the Eurasian three-toed woodpecker and NCBI reference sequences (NC_088452.1) were spread across the entire mitogenome or localized to a specific region, we calculated the number of base pair differences between the two in sliding windows of 1 kb.

### Phylogenetic analyses

The most read-abundant 16S rRNA sequence recovered from fresh sap rows was aligned with the seven newly generated and eight previously published mitogenomes of nine Swedish woodpecker species using mafft v7.526 [29]. A maximum-likelihood phylogenetic tree was constructed with iq-tree v2.3.6 [30], employing ultrafast bootstrap approximation (-B 1000) and the SH approximate likelihood ratio test (-alrt 1000) to assess node support, and visualized in Figtree v1.4.4 [31]. We constructed a second phylogeny based on 687 bp of the Cytochrome oxidase I (CO1) gene in order to understand conflicts in the full mitogenomic sequences of *Picoides tridactylus*. For the second phylogenetic analysis, we also included 20 publicly available COI sequences from the genus *Picoides* (accession numbers provided in S3 Table) that were downloaded from the BOLDSystems (Barcode of Life Data System) database [32] and the homologous loci from the 17 full mitogenome dataset, using mafft v7.526 [29]. We trimmed the full mitogenomes to the 687 bp region of COI using an in-house Python script. The second maximum-likelihood phylogenetic tree was constructed and visualized as described above.

## Results and discussion

**Fig 2:**
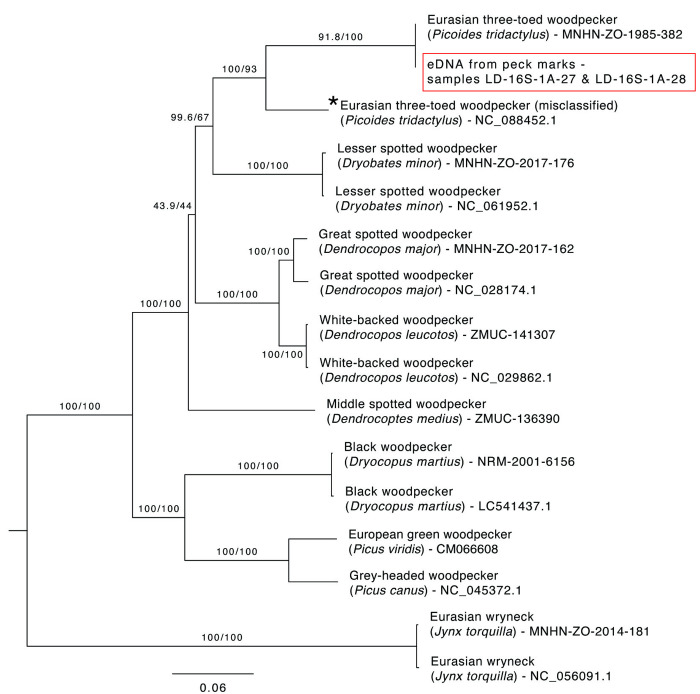
Phylogenetic tree of Swedish woodpecker species based on complete mitogenomes. Maximum likelihood phylogenetic tree based on seven newly generated and eight previously published mitogenomic sequences from nine woodpecker species currently present or historically recorded in Sweden, including the 16S rRNA eDNA sequence recovered from fresh peck marks (highlighted with a red box). The misclassified mitogenome for *P. tridactylus* is marked with an asterisk (*).

Out of the nine samples collected from fresh sap rows, PCR amplification of the expected size was successful in three samples (S1 Table). After sequencing, we generated between 212 and 8,276 paired-end reads per PCR product. Taxonomic assignment of reads with >85% identity resulted in the identification of two families, Picidae (woodpeckers) and Hominidae (human contamination), both of which were only identified with the Aves-16S-1AF/R primer pair. Two samples (LD_16S-1A-27 and LD_16S-1A-28) together contained the 15 Picidae sequences that all differed by only 1–2 bp from the most read-abundant sequence (S4 and S5 Table). This DNA sequence was identified as belonging to the woodpecker *Yungipicus canicapillus* from Asia with 89% identity (S5 Table). An NCBI BLASTn search confirmed this assignment and that this sequence (hereafter referred to as the eDNA sequence) matched the expected region of the 16S rRNA gene. The low percent identity to all woodpecker species available in the NCBI and EMBL databases was unexpected, as all eight extant woodpecker species in Sweden have complete mitogenomic sequences available in these databases. To further investigate the origin of our eDNA sequence, we generated new mitogenomes for seven Swedish woodpecker species, including the Middle Spotted woodpecker, which is extirpated in Sweden (S2 Table), and compared our data with eight published mitogenomes from the eight extant woodpecker species reported in Sweden (S3 Table). The newly obtained mitogenome for the Eurasian three-toed woodpecker (*Picoides tridactylus*) showed a 100% sequence match to our eDNA sequence but only shares 89.72% similarity with the NCBI reference sequence (NC_088452.1) (Fig 2). Furthermore, the divergence between the NCBI reference and newly generated mitogenome sequences is distributed across the entire length of the mtDNA, rather than being confined to a few regions (S1 Fig). This suggests that the observed variation is not the result of localized artifacts, such as chimera sequences. Instead, these two sequences represent two distinct mitochondrial genomes.

**Fig 3:**
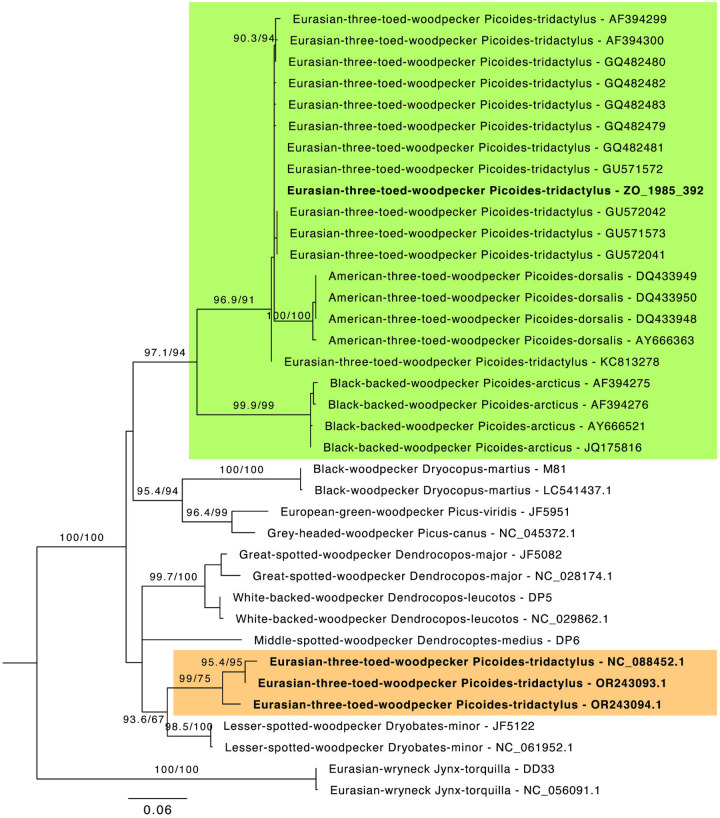
Phylogenetic tree of woodpecker species based on COI sequences. Maximum likelihood phylogenetic tree based on 687 bp of Cytochrome Oxidase I (COI) from 11 woodpecker species. Three *Picoides* species (including *P. tridactylus*, *P. dorsalis*, *P. arcticus*) are highlighted in green. The mitogenomic sequence for *P. tridactylus* generated here is in bold and highlighted green, whereas the three sequences available on NCBI are highlighted in bold and in orange.

Given that the dissimilarity between the two Eurasian three-toed woodpecker mitogenomes is beyond that expected from intraspecific variation otherwise observed in our woodpecker sequences (e.g., Fig 2), this raised the question as to which Eurasian three-toed woodpecker mitogenome best represents the genetic profile of the species in Sweden. We therefore generated a COI phylogeny of the genus *Picoides*, including 14 additional Eurasian three-toed woodpecker sequences, which revealed that all BOLDSystem *Picoides* sequences clustered into a monophyletic group alongside our newly generated sequences for the Eurasian three-toed woodpecker, whereas the NCBI reference sequences (including NC_088452.1 and two additional mitogenomes, OR243093-4, from the same study [33]) sit outside this group (Fig 3). This strongly indicates that the NCBI reference sequence for the Eurasian three-toed woodpecker, as well as OR243093-4, are misidentified and likely belong to *Dryobates* sp. This misidentification is possibly due to mistaken taxonomic identification or, more likely, a laboratory mix-up. The mitogenome sequence presented here best represents the Eurasian three-toed woodpecker. We therefore consider our eDNA sequence to be from the Eurasian three-toed woodpecker.

The Eurasian three-toed woodpecker, identified as the maker of the sap rows sampled here, is classified as Near Threatened in Sweden (Table 1). Monitoring for the presence of this species can aid in issuing recommendations on when and how logging should be conducted to minimize its impact on woodpecker habitats. One such effort was described by Ferry *et al.* [3], where the authors investigated whether three-toed woodpeckers create sap rows on deciduous trees, such as birches (*Betula* sp.). This was done by counting the number of observed sap rows and comparing them with the abundance of six woodpecker species across southern, central, and northern Sweden. The study found a weak (non-significant) correlation between the number of sap rows and the abundance of Eurasian three-toed woodpecker. However, the possibility that some sap rows may have been created by Great Spotted woodpeckers, which are also known to make such sap rows [34], could not be excluded. This hypothesis arose from the observation that a substantial number of trees with sap rows were located in areas where Eurasian three-toed woodpeckers are rarely observed, and Great spotted woodpeckers are frequently encountered, such as Scots pine-dominated, middle-aged production forests. The eDNA method used in this study shows potential to support such investigations and can serve as a complementary tool to traditional field-based methods for the monitoring of woodpecker species, particularly in cases where visual identification is uncertain or field surveys are logistically challenging. Moreover, the eDNA method is also cost-effective, with an estimated cost of 75–100 SEK (7–9 EUR) per sample, depending on the number of PCR replicates (1–3). This cost estimate includes DNA extraction using an automated robot (for higher throughput), PCR amplification for two regions, and sequencing ~10,000 reads on the Illumina NovaSeq X Plus platform. However, we note that for large-scale surveys and readily detectable species, traditional and more advanced acoustic monitoring methods are likely to remain the preferred approach, as they are easier to deploy in the field and require less laboratory processing compared to eDNA workflows.

One limitation of our study lies in its limited number of samples and success rate. For fresh sap rows, we observed amplification of the expected size in three out of nine samples, which were subsequently sequenced. Among these, woodpecker DNA was successfully identified in only two samples. This low success rate could be due to a combination of factors, including low DNA quantity, degradation, PCR inhibition, non-specific primers, and/or issues related to the sampling process. Moreover, in samples successfully amplified using primer pair 1, multiple read-abundant hits to human were observed, further indicating that the primers and PCR conditions used are not specific to woodpecker DNA (S4 and S5 Table). Future studies are needed to address these limitations through a more comprehensive sampling design and improved methodological approach, particularly by redesigning primer sequences to enhance specificity for woodpecker mtDNA and incorporating multiple primers in the same PCR reaction to increase the likelihood of detecting woodpecker. In the case of old sap rows, no avian 16S sequences were retrieved from any of the core samples. Despite these challenges, the study highlights the potential of eDNA as a non-invasive tool for species identification. With further optimization, this approach holds great promise for improving species identification and understanding woodpecker ecological interactions, which can inform conservation strategies and sustainable forest management practices.

## Supporting information

S1 Table Sample information and lab results. (XLSX)

S2 Table Sample information for the newly generated complete mitogenomes. (XLSX)

S3 Table Reference dataset used in the study. (XLSX)

S4 Table Sequencing stats and metabarcoding results. (XLSX)

S5 Table OBITools pipeline raw output. (XLSX)

S1 Fig Mitogenome comparison of the Eurasian three-toed woodpecker. Base pair differences between two mitogenomic sequences (MNHN-ZO-1985-382 and NC_088452.1) of the Eurasian three-toed woodpecker (*P. tridactylus*) calculated in 1 kb sliding windows.
(TIF)
