# Supplementary figures and images for: Environmental DNA from peck marks shows potential for non‑invasive monitoring of woodpeckers
Source: PLoS One. 2025 Aug 20;20(8):e0328831. doi: 10.1371/journal.pone.0328831 (PMC12367160; doi:10.1371/journal.pone.0328831)

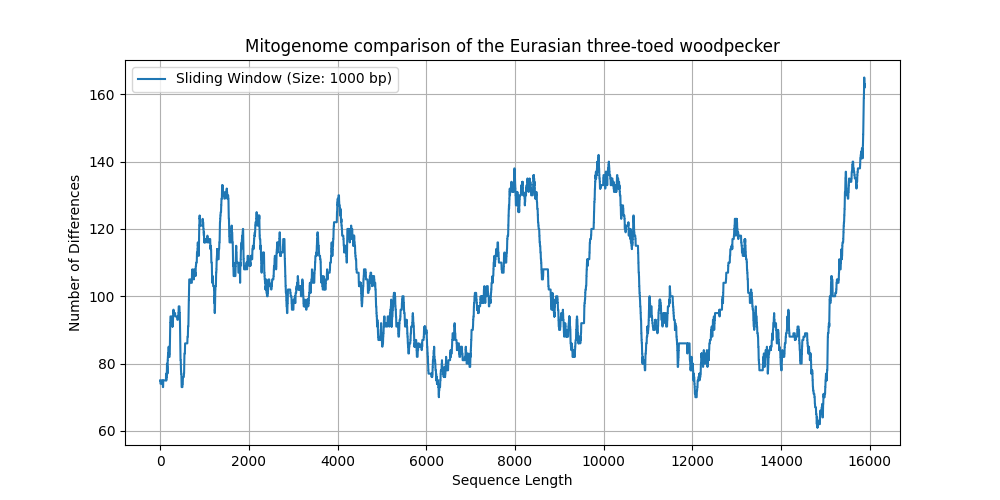

Supplement: S1 Fig — Base pair differences between two mitogenomic sequences (MNHN-ZO-1985-382 and NC_088452.1) of the Eurasian three-toed woodpecker (P. tridactylus) calculated in 1 kb sliding windows. (TIF) [file pone.0328831.s006.tif]
